# Public health and epidemiology of cancer in Colombia

**DOI:** 10.25100/cm.v49i1.3885

**Published:** 2018-03-30

**Authors:** Carolina Weisner

**Affiliations:** Directora. Instituto Nacional de Cancerología de Colombia, Bogota, Colombia

Understanding the epidemiology of cancer has been a relatively recent challenge for health systems of middle and low-income countries, since the predominant pathological profiles, up to the second half of the XX century, had been acute and communicable diseases and therefore cancer was not given sufficient visibility ^1^. The epidemiology of communicable diseases requires a specific epidemiological approach, with information that is almost in real time, or maximum up to one year; it also requires information based on the classification of the causal agent, with the primary objective of controlling epidemiological outbreaks ^2^. In contrast, the objectives of the epidemiological surveillance of cancer are: monitoring the behavior of different risk factors, estimating the population risk of developing the illness, -in a designated area and time-, as well as measuring the impact of interventions by analyzing survival and mortality ^2^. Since modifying the incidence or mortality by cancer requires interventions which imply a minimum of five years, generating information in cancer is generally done every five years. 

In high-income countries, the epidemiological transition towards the predominance of chronic illnesses began in the XVIII century ^3^. Since cancer started to become a significant public health problem in these countries, it was imperative to be able to measure cancer incidence. Different methodologies were used for this, such as surveys sent to physicians and passive registries that were not effective since we found that they only reported a third of the cases ^4^. This situation was very worrying because it makes it difficult to determine the population risk as well as the possibility to establish causal hypotheses in investigation ^4^. It was in 1946, when a Commission of international experts on the subject, suggested to the World Health Organization that they establish cancer registries with a standard methodology that was valid and reliable. This was the most significant precedent to form the International Association of Cancer Registries (IACR) in 1976 5.

Since then the International Agency for the Research on Cancer (IARC) has emphasized promoting a validated methodology such as population-based cancer registries (RCBP) 4. These registries constitute a strategy that allows us to collect data reliably on the incidence in a designated population which in addition allows us to make comparisons between countries ^6^. Frequently, we believe that with hospital and clinic registries it is possible to obtain data on incidence since the diagnosis of the patients that visit them is registered on these. However, the objective of these registries is mainly administrative and to register the clinical evaluation. For this reason, hospital registries are not useful to generate or provide measures on the appearance of cancer in a specific population and environment, precisely because it is not possible to define the population where the cases occur. Currently, 35% of all countries in the world have high quality RCBP to report cancer incidence and in Latin America only 22 % of countries count on these 2.

Colombia was a pioneer in the development of RCBP with the registry in Cali (RCPB-Cali), at the Universidad del Valle founded in 1962 under the boost of Pelayo Correa, an investigator focused on establishing causal hypotheses of stomach cancer and William Haenszel from the National Cancer Institute of the United States 7. Based on their creation, the RCPB-Cali has generated high quality information about cancer incidence, as the only one in the regional context, which has a population base of such a long trajectory 7-9.

The RCPB-Cali had a limited scope to demonstrate the reality of the entire country because Colombia has great geographical, demographical, social and cultural differences between its regions 10. Based on the expertise and trajectory of the RCBP in Cali, Pasto generated a second RCBP. Subsequently and with the additional support of the National Cancer Institute of Colombia (INC-Colombia) they created the RCBP of Bucaramanga, Barranquilla, Manizales, so that at the end of 2010 there were already five RCBP, four of which have been endorsed by the IARC, and its information circulated in the “*Cancer Incidence in Five Continents*” book 11. Other department registries in Antioquia, Cesar and Huila, have worked in generating information with difficulties in its implementation 12. Based on this information about municipal RCBP as well as information on mortality, the INC-Colombia applies the estimation methodology of the incidence used by the IARC as the main input to estimate cancer incidence in the different departments and for the country 13.

In addition, the RCBP allows us to generate survival information as the most important outcome to evaluate the comparative effectiveness of health systems regarding the control of the cancer 14. Global survival is also the most important outcome to evaluate the effectiveness of the treatments an health system. Undoubtedly, the comprehensive care of patients improves survival in that the cancer treatment requires a multidisciplinary look. 

This issue of Colombia Médica Magazine highlights the work done through the methodology based on the RCBP to obtain valid estimates of cancer incidence in a population. It is important to highlight this trajectory because since the year 2014, cancer incidence figures have been published in Colombia that have their source of information in the administrative database of the health system in Colombia (BDA-SSC for its initials in Spanish). These publications, which demonstrate the advantage of having national coverage, have generated controversies due to the magnitude of the differences in cancer incidence figures when compared to those generated by the RCBP 15. The sub-registry and the non-validation of the information generated by the BDA-SSC 16 are not useful to evaluate the population risk nor do they measure the impact policies, or the health system have had on controlling cancer in the country 17,18.

Based on the information of the RCBP and the mortality information from the DANE (National Administrative Department of Statistics); the INC-Colombia generates incidence estimates in the different departments of the country applying the methodology used by the IARC. The TIEE (initials in Spanish for incidence rates) for all cancers except skin cancer, were 151.5 in men and 145.6 in women, which contrasts with the rates in the United States of 347.0 in men and 297.4 in women.

Considering that survival rates is an outcome that must be evaluated for the health systems not only in terms of population but also institutionally, it shows survival data for patients treated for the first time for breast cancer and cervical cancer in the National Cancer Institute in the years 2007, 2010, and 2012. The 2-year survival rate of cervical cancer registered in the INC-Colombia is similar to the one registered in the Colombian and United States RPCC 14. On the contrary, the 24-month survival rate of breast cancer in the INC was 79.6% when for the five-year period of 2010- 2014 in the United States it reaches 90%.

Finally, and from a public health perspective, Colombia Medica Journal presents an analysis of oncological services carried out by the surveillance performed by the National Cancer Institute. In this sense, there is an analysis of services for adults as well as for children. Regarding the first group we find that in Colombia 87.9% of the provision of oncological services in Colombia is private in nature. Private companies as well as some insurers in Colombia open oncological services, especially for external consultation and chemotherapy, without guaranteeing comprehensiveness but prioritizing those services in which the use of cutting-edge technology represents a favorable business opportunity such as supplying expensive medications 19.

Likewise, and according to the articles presented, it is worrying that the Ministry of Health and Social Protection uses the same instrument to activate functional units for cancer treatment in adults and Childhood Cancer Comprehensive Care Units 20. It is clear that to guarantee the comprehensiveness for these two types of populations there must be a differential approach in which the fundamental variable is the frequency of the illness 19. It is not the same to guarantee the comprehensiveness for 1,312 new cases of cancer in children under 14 years of age than for the close to 62,000 cases of cancer in adults annually.

We expect that this issue of Colombia Medica contributes to promoting a unification of concepts relevant to the field of public health and epidemiological surveillance of cancer considering that it is the most important health challenge that we will have to face, moving forward, and therefore it is important to count on analysis instruments for health decision making. 
