# Supplementary material for: Public health and epidemiology of cancer in Colombia
Source: Colomb Med (Cali). 2018 Mar 30;49(1):13–5. doi: 10.25100/cm.v49i1.3885 (PMC6018826; doi:10.25100/cm.v49i1.3885)
Supplement: Related texts

# Editorial: Salud pública y epidemiología del cáncer en Colombia

Conocer la epidemiologia del cáncer ha sido un reto relativamente reciente para los sistemas de salud de los países de medianos y bajos recursos. Los perfiles patológicos predominantes, hasta la segunda mitad del siglo XX, habían sido las enfermedades agudas o transmisibles, por lo que no se daba una suficiente visibilidad al cáncer ^1^. 

La epidemiologia de las enfermedades trasmisibles requiere de un enfoque epidemiológico especifico, con información casi que en tiempo real, o máximo hasta con un año de atraso. Igualmente requiere de información basada en la clasificación del agente causal, con el objetivo primordial, de controlar los brotes epidémicos ^2^. En contraste, la vigilancia epidemiológica del cáncer tiene como objetivos: monitorear el comportamiento de los diferentes factores de riesgo, estimar el riesgo poblacional de desarrollar la enfermedad, -en un área y en un tiempo delimitados-, así como medir el impacto de las intervenciones mediante el análisis de la supervivencia y de la mortalidad ^2^. Dado que modificar la incidencia o la mortalidad por cáncer requiere de intervenciones que implican mínimo cinco años, la generación de información en cáncer se realiza en general de manera quinquenal.

En los países de altos ingresos, la transición epidemiológica hacia el predominio de enfermedades crónicas se inició en el siglo XVIII ^3^. Como el cáncer empezó a convertirse en un problema importante de salud pública en estos países, resultaba imperativo poder medir la incidencia de cáncer. Para ello se utilizaron diversas metodologías, como las encuestas enviadas a los médicos y los registros pasivos, que no lograron ser efectivas pues se encontró que éstas solo reportaban un tercio de los casos ^4^. Esta situación resultó ser muy preocupante porque hacía difícil determinar el riesgo poblacional así como la posibilidad de establecer hipótesis causales en investigación ^4^. Fue en 1946, cuando una Comisión de expertos Internacionales en el tema, sugirió a la Organización Mundial de la Salud el establecimiento de registros de cáncer con una metodología estándar que fuera valida y confiable. Este fue el antecedente más importante para la conformación, en 1976, de la Asociación internacional de registros de cáncer (IACR) ^5^.

Desde entonces la Agencia Internacional para la Investigación del Cáncer (IARC) ha hecho énfasis en promover una metodología validada como son los registros de cáncer de base poblacional RCBP) ^4^. Estos registros constituyen una estrategia que permite recoger datos de una manera confiable sobre incidencia en una población determinada que permite además hacer comparación entre los países ^6^. Con frecuencia se cree que con los registros de los hospitales y clínicas es posible obtener datos de incidencia pues en ellos se registran los diagnósticos de los pacientes que acuden a ellos. Sin embargo, el objetivo de estos registros es principalmente administrativo y de registro de la evaluación clínica. Por ello, los registros hospitalarios no son útiles para generar o proporcionar medidas de la aparición de cáncer en una población y un entorno definido, precisamente porque no es posible definir la población de donde surgen los casos. En la actualidad, el 35% de todos los países del mundo tienen RCBP de alta calidad para reportar incidencia de cáncer y en Latinoamérica solo el 22%de los países cuentan con ellos ^2^. 

Colombia fue pionera en el desarrollo de RCBP con el registro de Cali (RCPB-Cali), en la Universidad del Valle fundado en 1962 bajo el impulso de Pelayo Correa, investigador centrado en establecer hipótesis causales de cáncer de estómago y William Haenszel del Instituto Nacional de Cancer de Estados Unidos ^7^. A partir de su creación, el RCPB-Cali ha generado información de alta calidad sobre incidencia de cáncer, siendo el único en el contexto regional, que tiene una base poblacional de tan larga trayectoria ^7^^-^^9^.

El RCPB-Cali tenía un alcance limitado para mostrar la realidad de todo el país porque Colombia presenta grandes diferencias geográficas, demográficas, sociales y culturales entre sus regiones ^10^. Con base en la experticia y trayectoria del RCBP de Cali, Pasto generó un segundo RCBP. Posteriormente y con el apoyo adicional del Instituto Nacional de Cancerología de Colombia (INC-Colombia) se crearon los RCBP de Bucaramanga, Barranquilla, Manizales, de manera que a finales de 2010 ya existían cinco RCBP, cuatro de los cuales han sido avalados por la IARC, y su información divulgada en el libro “*Cancer Incidence in Five Continents*” ^11^. Otros registros departamentales en Antioquia, Cesar y Huila, han trabajado en la generación de información con dificultades en su implementación ^12^. Con base en los datos de los RCBP de base municipal así como de los datos de mortalidad, el INC-Colombia aplica la metodología de estimación de la incidencia utilizada por la IARC como insumo principal para hacer la estimación de la incidencia de cáncer en los diferentes departamentos y del país ^13^. 

Los RCBP permiten además generar información de supervivencia como el desenlace más importante para evaluar la efectividad comparativa de los sistemas de salud respecto al control del cáncer ^14^. La supervivencia global es igualmente el desenlace más importante para evaluar la efectividad de los tratamientos al interior de una Institución. Sin duda alguna la atención integral de los pacientes mejora la supervivencia por cuanto el tratamiento del cáncer requieres de una mirada multidisciplinaria. 

En este número de la Revista Colombia Médica se destacan los trabajos realizados mediante la metodología basada en los RCBP para obtener estimaciones válidas de la incidencia de cáncer en una población. Se requiere resaltar este hecho pues desde el año 2014, se han publicado en Colombia, cifras de incidencia de cáncer que tienen como fuente de información las bases de datos administrativas del sistema de salud en Colombia (BDA-SSC). Estas publicaciones, que presentan como ventaja tener una cobertura nacional, han generado controversias por la magnitud de las diferencias en las cifras de incidencia de cáncer cuando se comparan con las generadas por los RCBP ^15^. El sub registro y la no validación de los datos generados por las BDA-SSC ^16^ no resultan útiles para evaluar el riesgo poblacional y para medir el impacto que han tenido las políticas o el sistema de salud en el control del cáncer en el país ^17^^,^^18^.

Con base en los datos de los RCBP y los datos de mortalidad del DANE; el INC-Colombia genera estimaciones de incidencia en los diferentes departamentos del país aplicando la metodología utilizada por la IARC. Las TIEE para todos los cánceres excepto piel, fueron 151.5 en hombres y 145.6 en mujeres, que contrasta con las tasas en Estados Unidos de 347.0 en hombres y 297.4 en mujeres. 

Teniendo en consideración que la supervivencia es un desenlace que debe evaluarse no solo en términos poblacionales para los sistemas de salud sino de manera institucional, se presentan datos de supervivencia de las pacientes tratadas por primera vez en el Instituto Nacional de Cancerología por cáncer de mama y de cuello uterino en los años 2007, 2010, y 2012. La supervivencia a dos años al cáncer de cuello uterino registrada en el INC-Colombia es similar a la registrada en los RPCC-colombianos y en Estados Unidos ^14^. Por el contrario la supervivencia a 24 meses de cáncer de mama en el INC fue de 79.6% cuando para el quinquenio 2010-2014 en Estados Unidos ya llega al 90%.

Finalmente y desde una perspectiva de salud pública la revista Colombia Medica presenta en este número un análisis de los servicios oncológicos desde la vigilancia que sobre estos hace el Instituto Nacional de Cancerología. En este sentido, se hace un análisis de los servicios ofrecidos para la población adulta y pediátrica. En relación con los primeros, se determinó que el 87.9% de la oferta de servicios oncológicos en Colombia es de naturaleza privada. Las empresas privadas y algunos aseguradores ofertan servicios oncológicos, especialmente de consulta externa y de aplicación de quimioterapia, sin garantizar la integralidad en la atención al paciente. Ellos solo priorizan los servicios en donde el uso de tecnologías de punta garantiza una oportunidad de negocio propicia, como lo es el suministro de medicamentos de alto costo ^19^.

Igualmente y de acuerdo con los artículos presentados en este número, resulta preocupante que el Ministerio de Salud y Protección Social utilice el mismo instrumento para habilitar unidades funcionales para el tratamiento de cáncer en adultos y Unidades de Atención Integral de Cáncer Infantil ^20^. Es claro que para garantizar la integralidad para estos dos tipos de población se debe tener un enfoque diferencial en donde una variable fundamental es la frecuencia de la enfermedad ^19^. No es igual garantizar la integralidad para 1,312 casos nuevos de cáncer en menores de 14 años que para los cerca de 62,000 casos anuales de cáncer que se presentan en adultos.

Se espera que este número de Colombia Medica contribuya a promover una unificación de conceptos pertinentes al campo de la salud pública y de la vigilancia epidemiológica del cáncer teniendo en consideración que es el reto más importante en salud que, de cara al futuro, tendremos que enfrentar y por ello es importante contar con instrumentos de análisis para la toma de decisiones en salud.
